# The phenomenon of ragging: violence among university students in Sri Lanka

**DOI:** 10.1080/16549716.2026.2628362

**Published:** 2026-02-17

**Authors:** Ayanthi Wickramasinghe

**Affiliations:** Department of Women’s and Children’s Health, Faculty of Medicine, Uppsala University, Uppsala, Sweden

**Keywords:** hazing, harassment, young adults, South Asia, students, depression, mental health

## Abstract

**Background:**

Ragging is an initiation ritual in Sri Lankan universities where senior students harass newcomers. This practice leads to severe consequences such as depression, increased dropouts, and suicide, yet research on this issue remains scarce.

**Objective:**

This thesis aimed to explore ragging through the perspectives of students and university affiliates and to assess the prevalence of Major Depressive Disorder (MDD) among students.

**Methods:**

Study I was a cross-sectional survey (*n* = 623) among second- and third-year students from the Faculties of Medicine and Technology to determine the prevalence of ragging and its health consequences. Study II utilized focus group discussions (*n* = 17) with students to explore the social dynamics and motivations of ragging. Study III included focus group discussions (*n* = 7) and interviews (*n* = 11) with university staff to understand their attitudes towards ragging. Study IV surveyed second-year students (*n* = 637) from three faculties using the Patient Health Questionnaire to assess the prevalence of MDD.

**Results:**

Study I found that 59% of students experienced ragging, 54% reported health consequences, and most sought help from friends and family. Ragging prevalence varied by faculty and year of study. Study II revealed that ragging was used to establish power and address social inequalities. Study III identified themes of normalization, fear of reprisal, and resistance among staff. Study IV showed that 31% of students experienced MDD. MDD prevalence was associated with students’ ethnicity.

**Conclusion:**

Ragging is a pervasive issue with significant mental health consequences. Effective interventions require a multisectoral approach to create a safe and supportive university environment, enabling all students to thrive.

## Paper Context

**Main findings**: Ragging was experienced by 59%, 54% reported health consequences, and 31% experienced Major Depressive Disorder, while students used ragging to establish power and address social inequalities.

**Added knowledge**: This thesis provides new insight into ragging as a sociocultural and public health issue in Sri Lankan universities. The study highlights how societal inequalities, institutional weaknesses, and cultural norms sustain ragging, offering recommendations to promote safer and more supportive university environments.

**Global health impact for policy and action**: Ragging is a major public health and education concern in South Asia. Policies should integrate mental health support, violence prevention, and stronger enforcement of anti-ragging measures. A coordinated effort among universities, health authorities, and policymakers is essential to create safer, more supportive learning environments.

## Background

University entrance is usually a joyous milestone, symbolizing the start of an academic journey filled with new friendships and cherished memories. Yet, for some, this occasion is clouded by apprehension and fear due to the widespread harmful initiation practices like hazing and bullying in educational institutions globally. These practices, known by various names like ‘Hazing,’ ‘Bizutage,’ ‘Praxe,’ ‘Nollning,’ ‘Mopokaste,’ and ‘Ragging,’ serve as rites of passage intended to foster group cohesion in universities [1–4]. However, in recent years, these rituals have increasingly morphed into forms of abuse, causing psychological harm, physical injuries, and, unfortunately, even fatalities. Studies conducted across different countries have revealed prevalence rates, ranging from 37–78% within American universities and colleges [3], 48% among American high school students [5], and as high as 78% in Portuguese universities [6].

While initiation rituals vary in nature and severity, they often entail coercion and intimidation, with newcomers subjected to verbal, physical, and sometimes sexual abuse by their seniors [2,3,7]. These practices not only perpetuate existing power dynamics but also reflect broader societal issues related to race, gender, and socioeconomic status [8]. In the United States and Canada, hazing within fraternities and sororities often involves sexualized activities, excessive drinking, and dangerous initiation tasks [3,9]. Conversely, in Portugal, ‘Praxe’ is used to enforce conformity and integration among first-year students [2]. In Sri Lanka, ragging practices are seen as an opportunity to create a student group that is homogeneous [10,11].

‘Ragging’ is an initiation ritual practiced in South Asian universities like India, Pakistan, Nepal, Bangladesh and Sri Lanka [12–16]. In Sri Lanka it is defined as ‘any deliberate act by an individual student or group of students, which causes physical or psychological stress or trauma’ [17]. This can include psychological, physical, or sexual violence [12]. A 2002 study in Sri Lanka found varied responses to ragging, with some enjoying it while others considered it mental and physical trauma [18]. In another study among dental students, 50% reported harassment, mainly psychological [12]. Physical or sexual harassment was less common but still present, with 18% of male students experiencing sexual harassment [12]. In Pakistan, a study among six medical colleges revealed widespread bullying where 52% of the students had been bullied or harassed and 57% experienced verbal abuse [19].

Ragging in Sri Lanka is viewed as a longstanding university ‘subculture,’ perpetuated cyclically with changing roles between victims and perpetrators [20]. Traditionally, it involved harmless tasks but has evolved to include harmful practices like verbal abuse, physical assault, and sexual harassment [1,10]. Ragging often occurs within the same ethnic or gender groups, reflecting societal norms [11,21]. However, because interaction between genders is limited before entering university, ragging also becomes an opportunity for senior students to engage with juniors of the opposite sex [21].

This practice has detrimental effects, including permanent psychological damage, withdrawal, and altered personalities [10,12,22–24]. Studies in Brazil and Pakistan show links between ragging and depressive symptoms, while in Nepal, medical students found it to be highly stressful [23,25]. Ragging has led to severe injuries and even deaths in Sri Lanka [10]. Fear of ostracism prevents many victims from reporting ragging [26]. Those who do file complaints can be isolated, labeled as ‘anti-raggers,’ and excluded from university activities and social circles [21]. Studies suggest that many students see enduring ragging as a necessary sacrifice for acceptance, favor from seniors, and access to higher education [2,10,27].

## Study context

Sri Lanka, a country with a population of 21 million, boasts a 92% literacy rate and higher human development indicators compared to its regional counterparts. It is a diverse nation, comprising Sinhalese (75%), Tamils (11%), Moors (Muslims) (9%), and other ethnic groups. Official languages include Sinhala, Tamil, and English [28].

Educational reforms, particularly the introduction of universal free education in 1945, significantly altered Sri Lanka’s social landscape [29]. District quotas in universities diversified student populations compared withthe previous elite upper class students, leading to increased politicization and the emergence of socialist student unions [30]. However, limited university seats leave many qualified students unable to enroll, exacerbating disparities in infrastructure and academic quality [26].

Ragging is a criminal offense in Sri Lanka under the Prohibition of Ragging and other Forms of Violence in Educational Institutions Act, No. 20 of 1998, which carries severe punishment [31]. All universities have set up hotlines and mobile apps for incident reporting. Perpetrators are barred from campus and attending classes. New students learn about regulations, consequences of ragging, and support services during introductory programs. Despite all these efforts ragging incidents persist in universities.

## Depression among university students

Depression affects around 280 million people globally and is a major cause of disability, projected to become the primary cause of non-fatal burden of disease by 2030 [32]. Self-reported depression rates among individuals aged 15–24 years are approximately 19% according to a UNICEF study conducted in 21 countries [33]. Depression among young adults and students disrupts academic performance, social development, increases university dropouts, and is associated with substance abuse and suicide risk [34,35]. Studies report varying prevalence rates of depression among university students, ranging from 10% to 85%, with an average of 30.6% [36]. In Sri Lanka, depression is prevalent among university students, with emotional disorders reported as high as 63% among undergraduates [37–39]. Contributing factors include academic pressure, parental expectations, financial difficulties, and challenges entering the job market [37,40]. Ragging, in Sri Lankan universities, has also been linked to mental health disorders and suicides among students [12,37,40].

## Impact of the COVID-19 pandemic

The COVID-19 pandemic further disrupted education in Sri Lanka, with periodic closures and online learning becoming the norm. Students faced additional challenges, including financial strains and uncertainties about future employment prospects, alongside the mental health implications of the pandemic itself [41]. Sri Lanka experienced three significant COVID-19 surges, leading to multiple lockdowns and closures of educational institutions, including universities. They began reopening in July 2020 but faced periodic closures throughout 2021 due to ongoing waves of the virus. Education transitioned online, with limited in-person sessions for practicals and exams, and strict hostel rules to prevent infection [41,42].

Ragging poses significant health risks to students, requiring urgent action to address this public health issue. However, the lack of research and baseline data in Sri Lanka hinders effective efforts to combat ragging. Further studies are needed to understand the complex dynamics involved. Recognizing high levels of stress and anxiety among students, particularly at the University of Jaffna, there is a pressing need for exploratory research on depression to inform intervention strategies. Therefore, the overall aim was to investigate ragging in a Sri Lankan university to better understand its impact on students’ lives and mental health. The specific aims of the studies were:

1. To investigate the prevalence of different types of ragging, self‐perceived health consequences, help‐seeking behavior, and factors associated with the experience, among students.
2. To explore students’ perceptions on the phenomenon of ragging and to understand how ragging affects student life.
3. To explore the perceptions of staff and work-affiliated individuals on the phenomenon of ragging.
4. To estimate the prevalence of major depressive disorder (MDD) and other forms of depression, and to evaluate factors associated with MDD among students.

## Methods

A mixed-methods approach was used across the four studies in the PhD thesis. Table 1 summarizes the different methodologies used across the four studies.

**Table 1.** Overview of the methods for study I-IV.

	Study 1	Study II	Study III	Study IV
Design	Quantitative	Qualitative	Qualitative	Quantitative
Participants	2nd & 3rd year students from the Faculties of Medicine and Technology	Students from all the faculties	Staff and other work-affiliated individuals attached to the University	2nd year students from the Faculties of Management, Science and Medicine
Data collection	Cross sectional survey using a questionnaire (*n* = 623)	Focus group discussions (*n* = 17)	In-depth interviews (*n* = 11) and focus group discussions (*n* = 7)	Cross sectional survey using PHQ-9 questionnaire (*n* = 637)
Analysis	Descriptive statistics, bivariate and multivariate Regression analysis	Thematic analysis	Foucauldian discourse analysis	Descriptive statistics, bivariate and multivariate Regression analysis

## Study location

The University of Jaffna, located in the Northern Province of Sri Lanka, was an optimal setting for this study due to its unique ethnic diversity, with students representing all three major ethnic groups, unlike most other universities in the country that are predominantly Sinhalese. The study covered the main campus in Jaffna town and an offsite campus in the Kilinochchi district, housing the Faculty of Technology which was established in 2015.

## Participants and sampling

For Study I, second- and third-year students from the Faculties of Medicine and Technology were invited to participate. The study was conducted to compare student norms across the two faculties. The Faculty of Medicine was selected because it is long-established, with well-defined traditions and cultural norms, while the Faculty of Technology, established in 2016, represents a newer academic environment with evolving student norms. Second-year students, who may have experienced or participated in ragging, were included, along with third-year technology students, who constituted the first cohort without senior students. In study II, heads of departments informed students about the study, leading to a sample of 50 males and 58 females aged 21 to 25 from second- and third-year across all faculties in the University of Jaffna. In the III study, letters were sent to department heads to recruit lecturers and student counsellors for focus group discussions (FGDs) and semi-structured interviews. Participants included lecturers, student counsellors, university security, hostel wardens, and medical personnel treating students. In study IV, all the second-year students from the Faculties of Management Studies & Commerce, Science and Medicine were invited to participate in the study. These faculties were chosen because they included a diverse mix of students representing all major ethnic groups. They also had the largest student populations, ensuring broader representation of the overall university community. In contrast, other faculties did not reflect the same diversity, as their medium of instruction was Tamil which limited enrollment to a single ethnic group. Including second-year students ensured uniformity and excluded potential biases from final-year academic pressures. First-year students typically experience ragging during this period, therefore, were excluded from all the studies to avoid re-traumatization.

## Data collection

In Study I, questionnaires were distributed in English to medical students and translated into Sinhala and Tamil for technology students. Data collection took place in February 2019 for the medical faculty and September 2019 for the technology faculty, after compulsory lectures. For Study II, 17 FGDs were conducted among the students in English, Sinhala, and Tamil. FDGs consisted of 4–8 participants in each session lasting 45–60 minutes. In Study III, seven FGDs and 11 semi-structured interviews were conducted among the staff, in English and Tamil to explore group norms and dynamics related to ragging. Discussions and interviews followed a guide, involving 4–8 participants per FGD, lasting 45–60 minutes. For Study IV, Patient Health Questionnaires (PHQ-9) were distributed after exams or lectures to the students. The PHQ-9, available in English, Sinhalese, and Tamil, screened for depression.

## Data analysis

Study I presents descriptive characteristics of students, their experiences with ragging, perceived health consequences, and help-seeking behaviors. Study IV utilized the PHQ-9 questionnaire to assess depressive symptoms. Likert scale responses were categorized into severity levels, and descriptive statistics were presented. In both Study I and IV, Chi-squared tests and logistic regression were used to assess associations. In both studies, results are presented in two models: an unadjusted model followed by an adjusted model including significant factors from univariate analysis (*p* < 0.20). Odds ratios (OR) with 95% confidence intervals were calculated, and statistical significance was set at *p* < 0.05 using RStudio (version 3.5.2). In Study II each FGD was transcribed, translated, and back translated into English. Thematic analysis [43] was used to identify themes and subthemes. Similarly, Study III included transcription and translation of interviews and FGDs into English. Data analysis employed Foucauldian Discourse analysis [44,45] to identify repetitive patterns within the discourses.

## Theoretical frameworks

Three theoretical frameworks, Structural Violence [46], Intersectionality [47], and Social Dominance Theory [48], were integrated in Study II to elucidate the complexities of ragging. Since universities function as microcosms of the larger society, these frameworks together reveal how social structures, identities, and hierarchies interact across macro, meso, and micro levels to sustain the phenomenon. Galtung’s theory of structural violence [46] reflects what occurs at the macro level of society. According to Galtung, direct violence is the visible expression of underlying, invisible forms of institutionalized harm embedded within social systems, such as ragging. Structural violence operates through societal structures that perpetuate inequality and normalize harm, while cultural violence legitimizes these patterns by embedding them within accepted social values and traditions. This perspective helps explain how ragging, though destructive, has persisted across generations and has become perceived as a normalized ‘subculture’ within university life. Intersectionality [47] offers a framework to understand how overlapping identities such as gender, ethnicity, caste, class, language, and geographical origin shape individual experiences of privilege and marginalization. Within the university’s microcosm, these intersecting factors influence students’ beliefs, behaviors, and social positioning. The interaction of these identities not only defines each student’s lived experience but also determines their placement within various social groups, often shaped by the dominance of more powerful or privileged groups. Social Dominance Theory [48] explains how individuals and groups organize into hierarchies based on identity, forming ‘in-groups’ and ‘out-groups.’ Members of dominant groups maintain power and privilege, while those in subordinate groups experience control or exclusion. Within universities, this dynamic manifests when senior students assert authority over juniors or those perceived as belonging to an ‘out-group,’ reinforcing social hierarchies that mirror broader societal patterns.

**Figure 1.**
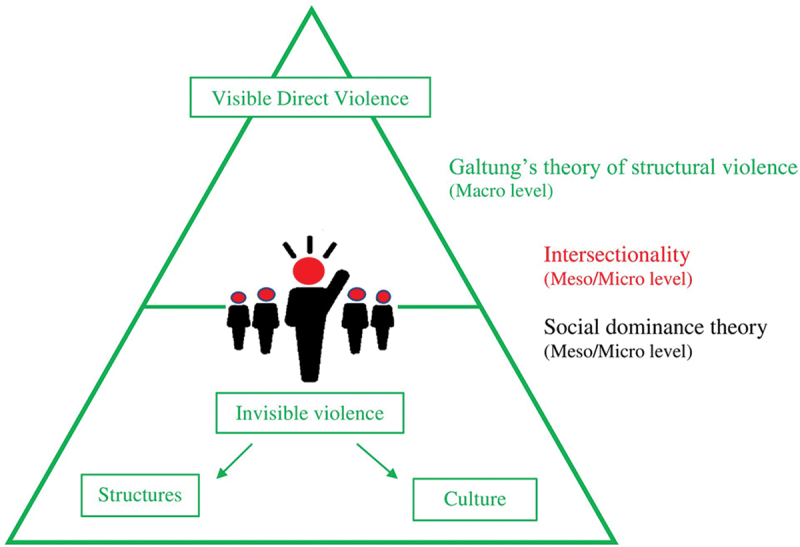
Integrated theoretical lenses (original creation).

Together, the theories of Structural Violence, Intersectionality, and Social Dominance provide a multilevel lens spanning the macro, meso, and micro dimensions of society to illuminate the systemic, cultural, and interpersonal complexities underlying the phenomenon of ragging (Figure 1).

In Study III, Foucauldian discourse analysis [44,45] combined with Bandura’s Moral Disengagement Theory [49] revealed how individuals navigate university conduct and moral responsibilities amidst ragging. Foucauldian analysis unpacked institutional power relations, critically analyzing staff narratives to reveal how university staff rationalized or justified their inaction towards ragging. Bandura’s theory illuminated the psychological mechanisms, such as moral justification and dehumanization, enabling staff to overlook or accept harmful practices, reflecting coping strategies within the university environment [49,50].

## Results

In Study I, from a total of 638 students, 623 completed the questionnaires in both faculties, giving a response rate of 91%. Amongst the medical students 118 (*n* = 149) of the second‐year students, and 128 (*n* = 138) of the third‐year students responded. While 190 (*n* = 200)

students in the second-year, and 187 (n = 196) students in the third-year responded in the Faculty of Technology. The descriptive statistics are shown in Table 2.

**Table 2.** Descriptive characteristics of the second- and third-year students from the faculties of medicine and technology.

Variable	Number of students (*N* = 623)	Experienced any type of ragging (*N* = 366*)	Did not experience ragging (*N* = 257*)	*p*-value**
**Sex**				
Female	331 (53%)	191 (58%)	140 (42%)	0.57
Male	292 (47%)	175 (60%)	117 (40%)	
**Age group**				
21 years	82 (13%)	51 (62%)	31 (38%)	**0.001**
22 years	266 (43%)	165 (62%)	101 (38%)	
23 years	222 (37%)	111 (50%)	111 (50%)	
≥24 years	45 (7%)	35 (78%)	10 (22%)	
**Ethnicity**				
Moors/Muslim	80 (13%)	49 (61%)	31 (39%)	0.25
Sinhalese	322 (52%)	179 (56%)	143 (44%)	
Tamil	221 (35%)	138 (62%)	83 (38%)	
**Father’s education**				
<11 years	158 (26%)	85 (54%)	73 (46%)	0.10
>11 years	453 (74%)	277 (61%)	176 (39%)	
**Father’s occupation**				
Self-employed	161 (27%)	101 (63%)	60 (37%)	0.45
Full-time employed	263 (43%)	149 (57%)	114 (43%)	
Unemployed/Irregular employment	185 (30%)	107 (58%)	78 (42%)	
**Mother’s education**				
<11 years	146 (23%)	75 (51%)	71 (49%)	**0.03**
>11 years	462 (75%)	285 (62%)	177 (38%)	
**Mother’s occupation**				
Employed	196 (32%)	122 (62%)	74 (38%)	0.28
Homemaker/Irregular employment	416 (68%)	240 (58%)	176 (42%)	
**Faculty**				
Medicine	246 (39%)	177 (72%)	69 (28%)	**<0.001**
Technology	377 (61%)	189 (50%)	188 (50%)	
**Year**				
Second year	308 (49%)	200 (65%)	108 (35%)	**0.002**
Third year	315 (51%)	166 (53%)	149 (47%)	

*Total sample size varies due to missing values. **Chi-squared were used to compare proportions. Values that are significant at the *p* < 0.05 level are shown in bold.

Among the students, 59% experienced at least one type of ragging. Emotional ragging was the most common form, with 40% of students experiencing at least one form of emotional/verbal ragging (Table 3). When stratified by sex, physical ragging was seen among 75% of male students (*p* > 0.05). No significant differences were observed by sex, ethnicity, or year of study for the types of ragging.

**Table 3.** Types of ragging experienced by the 2nd and 3rd year students from the faculties of Medicine and Technology.

Variable	Number (percentage of total sample) (*N* = 623)
**Emotional/verbal ragging**	
Yes	252 (40%)
No	371 (60%)
**Physical ragging**	
Yes	71 (11%)
No	552 (89%)
**Sexual ragging**	
Yes	80 (13%)
No	543 (87%)
**Any type of ragging**	
Yes	366 (59%)
No	257 (41%)

Students could respond positively to more than one statement.

Among the students who experienced any type of ragging, 54% reported at least one type of self‐perceived health consequences. Irritability/outbursts of anger were the most reported experience (33%), followed by upsetting memories (27%) and avoiding situations or activities (27%). Fifty-seven percent of students had sought some form of help, commonly from friends and family. The formal channels of help were underutilized.

The unadjusted analysis revealed that the maternal level of education, faculty and year of education were significantly associated with the experience of ragging among the students. Students who had mothers with more than 11 years of education had higher odds of experiencing any type of ragging compared to students who had mothers with lower than 11 years of education (UOR 1.52, 95% of CI: 1.04–2.21). Students belonging to the Faculty of Technology (UOR 0.39, 95% of CI: 0.27–0.55) and students in the third-year (UOR 0.60, 95% of CI: 0.43–0.82) had significantly lower odds of experiencing any type of ragging compared to the students in Faculty of Medicine and second-year students respectively. In the adjusted model maternal education was no longer associated with the experience of ragging (AOR 1.36, 95% of CI: 0.83–2.24). The lower odds of experiencing ragging among the students of the Faculty of Technology (AOR 0.44, 95% of CI: 0.29–0.63) as compared to the Faculty of Medicine remained significant in the adjusted model. Similarly, the decreased odds of being ragged in the third-year (AOR 0.67, 95% of CI: 0.46, 0.96) compared to the second-year was maintained after adjusting for other factors (Table 4).

**Table 4.** Factors associated with experience of any type of ragging among the second- and third-year students from the faculties of medicine and technology.

Variable	UOR	95% CI	AOR	95% CI
**Age**				
21 years	Reference	Reference	Reference	Reference
22 years	0.99	0.59, 1.65	1.04	0.60, 1.76
23 years	0.61	0.36, 1.02	0.68	0.39, 1.17
>24 years	2.13	0.95, 5.08	2.17	0.89, 5.72
**Father’s education**				
<11 years	Reference	Reference	Reference	Reference
>11 years	1.35	0.93, 1.94	0.97	0.59, 1.56
**Mother’s education**				
<11 years	Reference	Reference	Reference	Reference
>11 years	**1.52**	**1.04, 2.21**	1.36	0.83, 2.24
**Faculty**				
Medical	Reference	Reference	Reference	Reference
Technology	**0.39**	**0.27, 0.55**	**0.44**	**0.29, 0.63**
**Year**				
Second year	Reference	Reference	Reference	Reference
Third year	**0.60**	**0.43, 0.82**	**0.67**	**0.46, 0.96**

UOR-unadjusted odds rations, AOR-adjusted odds ratio; CI-confidence interval; Values that are significant at *p* < 0.05 level are shown in bold.

**Figure 2.**
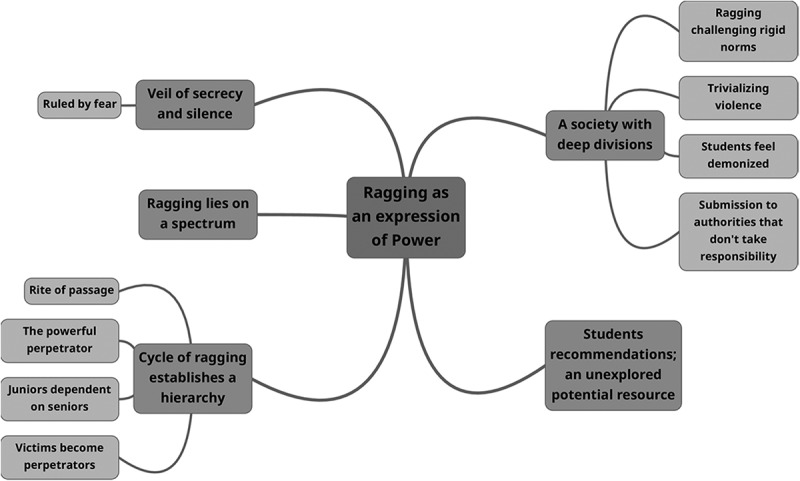
Main theme and subthemes derived from the analysis of student focus group discussions.

The main themes identified in Study II were: Veil of secrecy and silence, Ragging lies on a spectrum, Cycle of ragging establishes a hierarchy, A society with deep divisions and Student recommendations an unexplored potential with Ragging as an expression of power, as the overarching theme (Figure 2).

The findings revealed how students used ragging as an expression of power, to show dissatisfaction towards social inequalities, as a forum to demonstrate power, and to initiate order as well as a way to facilitate bonds between students within the university, acting as a mirror of the larger society. Students also trivialized the violence related to ragging and accepted it as a part of the university subculture despite being aware of the dire consequences. There was a cyclical nature to ragging where victims become perpetrators the following year. Although, students often felt unheard they could offer solutions to stop this practice.

The three main discourses found in Study III reflected the context: Ragging as normal and necessary, Insecurity and fear of reprisal, and Voices of resistance. Due to the inverted power dynamic within the University, where students wielded more power, study participants often felt unsupported and unsafe, causing them to adapt their moral compasses according to the mechanisms of moral disengagement in order to work and survive in this environment. By adjusting their moral compasses to tolerate the status quo, lecturers and work-affiliated individuals turned away from their obligations to do the right thing due to their powerlessness by turning a blind eye towards ragging.

In Study IV, from 687 second-year students across the faculties of Management Studies & Commerce, Science, and Medicine, 674 responded to the questionnaires, resulting in a 98% response rate. However, due to incomplete questionnaires, only 637 students were included in the study. The descriptive statistics are shown in Table 5.

**Table 5.** Descriptive characteristics of the students from the faculties of management studies & commerce, science and medicine.

Variable	Number of students (*N* = 637)	Students without MDD (*N* = 446)	Students with MDD* (*N* = 191)	*P* value**
**Sex**				
Female	378 (59%)	271 (72%)	107 (28%)	0.30
Male	259 (41%)	175 (68%)	84 (32%)	
**Age**				
<22 years	124 (19%)	93 (75%)	31 (25%)	0.09
23 years	226 (36%)	166 (73%)	60 (27%)	
24 years	178 (28%)	113 (63%)	65 (37%)	
>25 years	109 (17%)	74 (68%)	35 (32%)	
**Ethnicity**				
Moor/Muslim	46 (7%)	39 (85%)	7 (15%)	**0.02**
Sinhalese	290 (46%)	191 (66%)	99 (34%)	
Tamil	301 (47%)	216 (72%)	85 (28%)	
**Father’s schooling**				
<11 years	231 (36%)	153 (66%)	78 (34%)	0.14
>11 years	406 (64%)	293 (72%)	113 (28%)	
**Mother’s schooling**				
<11 years	199 (31%)	130 (65%)	69 (35%)	0.09
>11 years	438 (69%)	316 (72%)	122 (28%)	
**Faculty**				
Management^#^	305 (48%)	204 (67%)	101(33%)	0.25
Science	202 (32%)	148 (73%)	54 (27%)	
Medicine	130 (20%)	94 (72%)	36 (28%)	

*MDD - Major Depressive Disorder (PHQ-9 score ≥10). **Chi-squared was used to compare proportions. Values that are significant at the *p* < 0.05 level are shown in bold. ^#^Management Studies & Commerce faculty.

From the three faculties, 31% of the students reported experiencing MDD. Students from the Faculty of Management Studies & Commerce reported having experienced the highest proportion of MDD (15%). Symptoms of mild depression were reported by 39% of the students. Therefore 70% of the students had experienced some form of depression ranging from mild to severe.

In the unadjusted analysis, ethnicity was significantly associated with MDD (Table 6). Students who belonged to the Sinhalese ethnicity were at higher odds of experiencing MDD compared to the students who were ethnically Moor/Muslim (UOR 2.90, 95% of CI: 1.33–7.30). In the adjusted model, ethnicity continued to be associated with MDD, where Sinhalese students sustained increased odds of experiencing MDD compared to Moor/Muslim students (AOR 2.87, 95% of CI: 1.30–7.25)

**Table 6.** Factors associated with major depressive disorder (MDD) among the students from the faculties of Management studies & Commerce, Science and Medicine.

Variable	UOR	*P* value (95% CI)	AOR	*P* value (95% CI)
**Sex** Female Male	Reference 1.22	Reference 0.16 (0.86–1.71)	Reference 1.23	Reference 0.41 (0.80–1.59)
**Ethnicity** Moor/Muslim Sinhalese Tamil	Reference **2.90** 2.19	Reference **0.01 (1.33–7.30)** 0.06 (0.10–5.53)	Reference **2.87** 2.11	Reference **0.01 (1.25–6.93)** 0.07 (0.97–5.44)
**Father’s schooling** <11 years >11 years	Reference 0.76	Reference 0.06 (0.53–1.07)	Reference 0.91	Reference 0.58 (0.57–1.47)
**Mother’s schooling** <11 years >11 years	Reference 0.73	Reference 0.06 (0.51–1.04)	Reference 0.76	Reference 0.24 (0.47–1.23)
**Faculty** Management^#^ Science Medical	Reference 0.74 0.77	Reference 0.42 (0.50–1.09) 0.09 (0.49–1.21)	Reference–	Reference–

UOR-unadjusted odds ratio, AOR-adjusted odds ratio; CI-confidence interval; Values significant at *p* < 0.05 level are shown in bold. ^#^Management Studies & Commerce faculty.

## Discussion

This thesis aimed to investigate and gain a deeper understanding of the phenomenon of ragging in Sri Lankan universities from diverse perspectives. Despite its known negative consequences, ragging persists with increasing severity each year, impacting not only student victims but also university lecturers, staff, families, and the wider community. Our findings reveal that ragging continues at the University of Jaffna, both openly and covertly, due to various individual and communal factors within the university and society. The university staff are left struggling to cope with the detrimental effects of ragging, as well as the impact on the mental health of students.

## The phenomenon of ragging in the university of Jaffna

The thesis reveals how students use ragging to assert power in what they believe to be an unjust and unequal society marked by rigid social norms and hierarchical systems, where their voices go unheard [10,46,51]. Sri Lankan youth, disenfranchised by socioeconomic divides and power imbalances, use ragging to challenge societal norms and equalize social disparities [11,28,48]. This dissatisfaction can turn into direct violence [46] which manifests as ragging. Students at the University of Jaffna intersect across diverse backgrounds but are often subjected to hierarchical systems perpetuated by senior students through ragging. Despite this, sociodemographic factors like ethnicity, gender, and parental education have not been significantly linked to violence such as ragging [52]. Despite this, Sri Lankan youth often feel deprived due to the societal divisions of socioeconomic class, caste, ethnicity and gender, and the uneven distribution of power [51,53]. Furthermore, the urban, English-speaking, middle class attain pivotal positions in the private sector while the rest have to settle for the limited jobs in the government and public sector [11,54]. According to Gamage et al. [28] ragging has been used as a way to disrupt the social norm by bringing down the privileged to a more even level. The findings from the FGDs with students demonstrated, a similar occurrence that aligns with the Social dominance theory [48], where the students of the University of Jaffna, from various socioeconomic classes, ethnicities, castes and genders intersect [47,55] and are regrouped according to a system where the juniors are ragged by the seniors. The students claimed that ragging was used as a method of overcoming social hierarchies and ‘equalizing’ all students, which they believed gave a voice to the socially deprived and marginalized. The idea that ragging helped to bring all students to the same level and maintain uniformity was a sentiment common to lecturers and other individuals affiliated with the university. In the quantitative study, sociodemographic factors such as ethnic background, gender and father’s and mother’s education, which were used as a proxy for socioeconomic status, were not significantly associated with ragging. Nonetheless, research suggests that peer violence is more accepted among youth from the lower socioeconomic levels [52]. A multicounty study [56] and a study from Colombia [52] indicated that large disparities among individuals led to increased violence among students, which in turn contributes to practices such as ragging.

Ragging was prevalent at the University of Jaffna, affecting more than half of the students. Similar rates were reported in a study at a Sri Lankan dental faculty [12], as well as in studies on bullying in Pakistan, ragging in Nepal, and hazing in Portugal, with prevalence rates ranging from 52% to 78% [6,14,19]. However, the actual prevalence could be higher, as students often conceal incidents due to secrecy and fear of reprisal from seniors [4,22].

Students in Sri Lankan universities experienced various forms of ragging within the university ‘subculture,’ ranging from mild activities like singing songs for new entrants to more severe behaviors involving emotional/verbal, physical, and sexual violence [20,21]. Although some junior staff at Jaffna University viewed ragging as ‘normal and necessary,’ believing it to be a rite of passage, other studies have recognized its harmful effects [10,11,21]. A report on eight state universities in Sri Lanka indicated that verbal ragging affected 51% of students, with 34% experiencing psychological, 24% physical, and 17% sexual ragging [21]. Emotional/verbal ragging was particularly common at the University of Jaffna, where seniors often engaged in shouting, punishment, and threats against juniors which was in line with other studies [12,13,23].

Physical ragging was less prevalent among our participants and in other Sri Lankan studies [12,57], contrasting with studies in the USA that reported severe injuries and deaths due to hazing [9,27]. The lower reported rates of physical violence may stem from students normalizing or trivializing violence, influenced by society’s dismissive nature toward violence [58]. A national youth survey conducted in 2013 in Sri Lanka demonstrated that 3.7% males and 1.5% females had been involved in fights, that required medical treatment within the last 12 months [58]. Recent reports indicate that violent behavior among youth is on the rise globally [59] as well as in Sri Lanka [60–62]. Sri Lanka’s own history of violence, including insurgencies and civil war [30], is also a contributory factor. This normalization of violence has impacted societal attitudes toward aggressive behaviors, potentially rendering practices like ragging to be seen as harmless [10]. Similar dismissive attitudes toward violence were observed in American studies on hazing, where students often downplayed harmful experiences [3,4].

Sex is a taboo subject rarely discussed in Sri Lanka [21], yet students disclosed experiencing milder forms of sexual ragging such as unwanted comments. Global studies indicate sexual violence during initiation practices, with some studies reporting high prevalence rates [12,27,63]. Serious incidents of sexual ragging have occurred in Sri Lankan universities and some have been reported in local newspapers [20,64,65]. In a context where sex education is lacking and traditional societal norms emphasize chastity and modesty [21], university students may hesitate to discuss sexual ragging due to embarrassment or fear of stigmatization.

Students from the longstanding medical faculty experienced higher victimization rates compared to those from the newly established technology faculty, attributed to the time needed to establish student norms. Ragging is typically conducted by senior students within each faculty [11,21], and the absence of senior students in the new Faculty of Technology should has reduced incidents of ragging among these students. However, third-year students in this faculty could have been targeted by seniors from other faculties sharing the premises. The isolated location of the Faculty of Technology provides opportunities for ragging, while the longstanding medical faculty, situated centrally, has well-established cultural norms including ragging practices [21].

Study participants revealed that ragging served as a means for seniors to exert power, and control juniors. This cyclical nature of ragging led former victims to anticipate their turn as seniors, often perpetuating harsher ragging practices [21]. Senior students used ragging to channel frustrations related to social inequalities, inferiority complexes, and personal jealousies [10,11,51]. Initiation practices like ragging are characterized by abuse of power, coercion, and domination, according to global studies [3,6,22].

### Health consequences and help-seeking behavior among the students

The findings revealed that 54% of students reported non-somatic health consequences such as upsetting memories, irritability, loneliness, and insomnia, aligning with other studies [12,23,25]. Consistent with previous research, ragging contributes to depression and withdrawal from university; however, this study further highlights that these effects are reinforced by institutional neglect, normalization of violence, and lack of adequate mental health support within universities [20,36]. These factors, together with cultural acceptance of hierarchical control, intensify the psychological toll on students and perpetuate the cycle of ragging across cohorts.

Despite available reporting methods, only half of the students who experienced ragging sought help, mostly relying on family and friends [3,66]. Many students view ragging as a bonding experience and part of university life, hindering reporting and help-seeking behavior [14,18]. This reluctance is fueled by distrust in the system, where perpetrators are shielded and rarely punished, leaving victims disillusioned with university administrations [57]. Students also fear ostracization and view enduring ragging as a required sacrifice for limited educational opportunities [53].

## Ragging as a gendered phenomenon

Gender differences in ragging and hazing reflect broader social and cultural inequalities [8,12,27]. While the quantitative study did not find a significant association between gender and ragging, other research shows that male students often experience more physical violence, whereas females endure psychological forms [5,8,15,19]. These contrasting experiences stem from Sri Lanka’s entrenched patriarchal norms, where men are expected to display dominance and toughness, while women are expected to embody modesty and obedience [8,27].

Within this societal framework, male dominance and female submission are reinforced, perpetuating unhealthy gender norms that harm both sexes [21]. For male seniors, ragging provides an outlet to assert authority and display their masculinity [11,21]. Patriarchal ideas such as Læjja-baya (shame and fear) are used to regulate women’s behavior through ragging, reinforcing ideals of modesty and chastity [21]. Study participants described how senior females enforced these expectations by monitoring how juniors dressed and behaved to prevent them from becoming targets of ‘shame and ridicule’ [21]. Although Sri Lankan society traditionally expects women to be submissive and home-bound, the growing presence of women in universities challenges these norms [10,21].

Despite this progress, few women attain senior academic, corporate, or political positions due to ongoing barriers such as inadequate maternity leave and childcare support [67]. These disparities in higher education and employment mirror the broader struggle for gender equity in Sri Lanka [68].

## Perceptions of students and university staff on ragging

Both students and staff at the University of Jaffna viewed ragging as fostering group cohesion and leadership development, a sentiment echoed in other studies [3,4]. Students from rural backgrounds especially saw benefits in personal growth, overcoming shyness, and improving communication [10,21]. Ragging was also seen as reinforcing societal roles and norms, instilling obedience to seniors, which is crucial in Sri Lankan hierarchical culture [21]. Lecturers believed that ragging taught students to follow rules and behave appropriately, linking to concepts of moral disengagement [49,69,70].

Participants from both lecturers and students claimed that ragging ensured adherence to societal roles and norms. In Sri Lankan society, respecting seniors is crucial [11,21]. Students felt juniors must obey seniors to succeed in life. Lecturers shared this view, believing ragging teaches rule-following and appropriate behavior. These findings align with the concept of ‘Moral Disengagement,’ [49] influenced by social norms, making ragging more acceptable. Additionally, lecturers noted that unruly student behavior resulted from a lack of ragging, leading to consequences for them, illustrating evasion of moral responsibility due to self-interest [49].

However, perceptions of ragging’s intensity varied. While students argued it had become harmless bonding, staff believed it had escalated over time, aligning with global trends showing a shift towards more violent forms [1–3]. This escalation parallels gang initiations, where violence is used for acceptance [71]. Some research suggests severe initiations can hinder group cohesion, contrary to popular belief [72], highlighting potential drawbacks of escalating ragging practices.

### Inversion of power within the university

Lecturers and students at the University of Jaffna expressed mutual distrust and dissatisfaction. The lecturers felt that the students lacked trust and did not respect them, which the students also mentioned during their interviews. Students felt that they did not receive the support they required and that staff were insensitive towards their needs [73]. Lecturers also perceived that the students felt that some of the lecturers were incompetent and attributed student aggression to disappointment with teaching methods and lack of unity among staff [57,74]. Outdated pedagogy and top-down teaching styles inhibit dialogue and knowledge exchange, exacerbated by recruiting inexperienced graduates as lecturers [74]. The staff felt that these factors could be the cause of studentaggression that has led to instances where the students have threatened them, which has made the staff more cautious when intervening in ragging incidents. The discord among staff contributes to structural issues within the institution and the failure of certain anti-ragging interventions [73,75] promotes an environment where students may resort to violence like ragging [30]. Furthermore, these inadequacies and fragmentation within the university have led to a frail power structure, and an opportunity for the students to seize power [26,51]. This inversion of power has caused the staff to adapt to survive within this environment, by potentially shifting moral standards through mechanisms like Moral Disengagement [49].

## Major depressive disorder among the students

The initial studies highlighted the urgent need for mental health exploration among University of Jaffna students. Over half of those in the ragging quantitative study reported non-somatic health issues like insomnia and irritability, potentially linked to serious mental disorders [15,24,76]. Qualitative findings revealed additional stressors including academic pressure, financial strain, insecurity, and homesickness. The civil war’s lingering effects may contribute to unresolved aggression [77], underscoring mental health support gaps.

Second-year students in Management Science & Commerce, Science, and Medicine faculties showed over a third suffering from Major Depressive Disorder (MDD) [76]. Comparison with University of Colombo data revealed higher rates [37], while studies conducted at University of Ruhuna in Sri Lanka also noted high depressive symptoms among medical and non-medical students [38,78]. International figures vary, with higher rates in low- and middle-income countries possibly due to stigma and limited access to mental health care [24,79].

Despite elevated depression rates, underreporting likely persists due to Sri Lanka’s mental health stigma rooted in historical isolation of patients. The country’s low psychiatrist-to-population ratio exacerbates access challenges, though strides are being made to improve mental health care and reduce stigma [80,81].

## Strengths and limitations

The overall strengths of this research include a comprehensive approach covering diverse faculties, academic years, and ethnic groups, supported by high response rates and the use of participants’ preferred languages. The involvement of a diverse research team and consistent data collection methods enhanced data quality and reliability. These factors provided rich, varied insights into the phenomenon of ragging within the university context.

The cross-sectional design limits causal interpretation. Ragging’s sensitivity likely led to underreporting, meaning actual prevalence may be higher. The study’s geographic focus on the University of Jaffna may limit generalizability, as its post-war context and ethnic composition differ from other universities. COVID-19 restrictions also influenced data collection, with periodic campus closures and online learning possibly affecting students’ experiences and responses.

Additionally, social desirability bias and the potential withholding of information to protect institutional reputation could have influenced responses. Lack of baseline mental health data prior to university enrollment limits interpretation of mental health findings.

## Conclusion

This thesis highlights ragging as a complex issue at the University of Jaffna, reflecting deeper societal problems rooted in entrenched sociocultural norms and violence. Ragging reflects power dynamics and attempts to equalize students, revealing broader systemic weaknesses within Sri Lanka’s education system and university administration. Fragmentation and lack of institutional support have led to a student-driven power shift, forcing staff to adapt by morally disengaging from student matters like ragging.

The high prevalence of MDD among students underscores the psychological toll of ragging, compounded by academic pressure, financial strain, war-related trauma, and separation from home. Addressing these challenges requires comprehensive institutional and policy responses.

Universities should strengthen counselling and mentorship services, promote peer support groups, and provide mental health and recreational programs to help students cope with stress. Staff accountability and leadership training on conflict resolution and violence prevention should be enhanced, alongside regular awareness programs for both staff and students on the harmful consequences of ragging. Security measures and enforcement of anti-ragging laws must also be reinforced.

Furthermore, fostering trust and positive interaction between students, staff, and administrators is essential to create a culture of respect and safety. A multisectoral, participatory approach involving university authorities, the University Grants Commission, policymakers, student unions, and civil society is vital to eliminate ragging and ensure a supportive educational environment for all students.
